# Increased Adiposity, Dysregulated Glucose Metabolism and Systemic Inflammation in Galectin-3 KO Mice

**DOI:** 10.1371/journal.pone.0057915

**Published:** 2013-02-22

**Authors:** Jingbo Pang, Davina H. Rhodes, Maria Pini, Rand T. Akasheh, Karla J. Castellanos, Robert J. Cabay, Dianne Cooper, Mauro Perretti, Giamila Fantuzzi

**Affiliations:** 1 Department of Kinesiology and Nutrition, University of Illinois at Chicago, Chicago, Illinois, United States of America; 2 Department of Pathology, University of Illinois at Chicago, Chicago, Illinois, United States of America; 3 The William Harvey Research Institute, Barts and the London School of Medicine, Queen Mary University of London, London, United Kingdom; University of Tor Vergata, Italy

## Abstract

Obesity and type 2 diabetes are associated with increased production of Galectin-3 (Gal-3), a protein that modulates inflammation and clearance of glucose adducts. We used Lean and Diet-induced Obese (DIO) WT and Gal-3 KO mice to investigate the role of Gal-3 in modulation of adiposity, glucose metabolism and inflammation. Deficiency of Gal-3 lead to age-dependent development of excess adiposity and systemic inflammation, as indicated by elevated production of acute-phase proteins, number of circulating pro-inflammatory Ly6C^high^ monocytes and development of neutrophilia, microcytic anemia and thrombocytosis in 20-week-old Lean and DIO male Gal-3 KO mice. This was associated with impaired fasting glucose, heightened response to a glucose tolerance test and reduced adipose tissue expression of adiponectin, Gal-12, ATGL and PPARγ, in the presence of maintained insulin sensitivity and hepatic expression of gluconeogenic enzymes in 20-week-old Gal-3 KO mice compared to their diet-matched WT controls. Expression of PGC-1α and FGF-21 in the liver of Lean Gal-3 KO mice was comparable to that observed in DIO animals. Impaired fasting glucose and altered responsiveness to a glucose load preceded development of excess adiposity and systemic inflammation, as demonstrated in 12-week-old Gal-3 KO mice. Finally, a role for the microflora in mediating the fasting hyperglycemia, but not the excessive response to a glucose load, of 12-week-old Gal-3 KO mice was demonstrated by administration of antibiotics. In conclusion, Gal-3 is an important modulator of glucose metabolism, adiposity and inflammation.

## Introduction

Obesity and its associated co-morbidities are among the most problematic health conditions modern societies have to deal with [1]. Obesity, particularly accumulation of visceral adipose tissue (VAT), is characterized by chronic inflammation that likely plays an important role in increasing the risk of chronic pathologies [1]. Individual differences in the degree of adiposity, the immune and inflammatory response, the ability of the organism to handle oxidative stress as well as composition of the gut microbiota are important factors in the development of obesity-associated co-morbidities [2].

Galectin-3 (Gal-3), a member of the galectin family, has been widely studied for its involvement in inflammatory responses [3]. Production of Gal-3 is highly increased during inflammation in both humans and animals and Gal-3 exerts pro-inflammatory effects under a variety of conditions [3]. However, the effect of Gal-3 deficiency on inflammation remains controversial. In fact, although Gal-3 KO mice exhibit decreased inflammatory responses in models of peritonitis as well as bacterial, parasitic and prion infection [3], they demonstrate exacerbated sensitivity to endotoxin [4]. Moreover, Gal-3 KO mice subjected to diet-induced atherosclerosis or diabetes-associated kidney damage experience increased oxidative stress and inflammatory responses, leading to more severe pathology [5]–[8]. The increased pathology of Gal-3 KO mice in these models may be secondary to the ability of Gal-3 to act as a scavenger for advanced glycation and lipoxidation end-products, with data demonstrating elevated levels of these adducts in Gal-3 KO mice, particularly when fed an atherogenic diet [6], [9]. In agreement, the increased circulating levels of Gal-3 observed in patients with Type 2 Diabetes are negatively correlated with glycated hemoglobin (HbA1c), suggesting a possible protective role for Gal-3 in the setting of hyperglycemia [10]. On the other hand, controversial results have been published on the effect of Gal-3 deficiency in models of hepatic steatosis/inflammation, with studies indicating either protection or increased disease severity in Gal-3 KO mice [9], [11], [12]. However, there is agreement that Gal-3 KO mice demonstrate elevated hepatic expression of peroxisome-proliferator-activated receptor γ (PPARγ), suggesting that Gal-3 participates in the regulation of fatty acid and glucose metabolism in the liver [9], [12].

Galectin-3 has also been studied in the context of obesity. In adipose tissue, Gal-3 is expressed by both adipocytes and infiltrating macrophages [13]. Evidence indicates that circulating levels and adipose tissue production of Gal-3 are elevated in obesity in both humans and experimental animals, with higher expression in VAT compared to subcutaneous adipose tissue (SAT) [10], [13], [14]. Moreover, Gal-3 promotes preadipocyte differentiation *in vitro* [15], suggesting that increased Gal-3 may help drive the expansion of adipose tissue in obesity.

In order to more clearly understand the role of Gal-3 in obesity and its associated metabolic and inflammatory consequences, in the present study we investigated the effect of Gal-3 deficiency using the model of high fat diet (HFD)-induced obesity (DIO) in mice.

## Results

### Increased adiposity in Gal-3 KO mice

**Figure 1:**
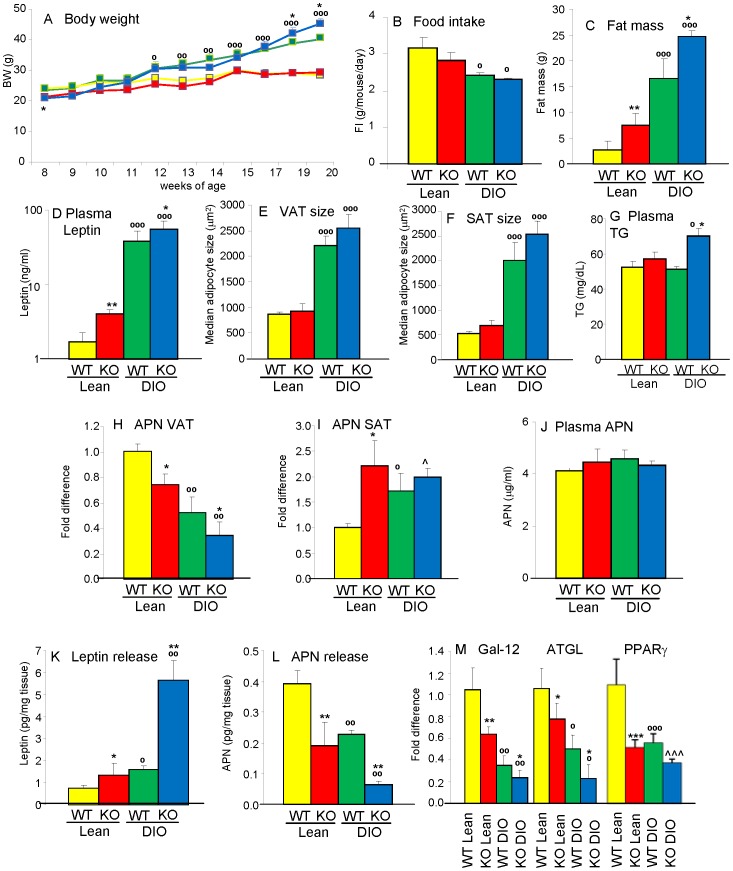
Increased adiposity in Gal-3 KO mice. Parameters of adiposity were evaluated in Lean WT (yellow), Lean Gal-3 KO (red), DIO WT (green) and DIO Gal-3 KO (blue) mice. Body weight (**A**) was evaluated weekly. Food intake (**B**) was evaluated weekly and average daily food intake per mouse is shown. Absolute fat mass by DXA (**C**), plasma leptin (**D**) and APN (**J**) levels as well as median adipocyte size (µm^2^) in VAT (**E**) and SAT (**F**) were measured at week 20, together with plasma TG levels (**G**). Expression of mRNA for APN in VAT (**H**) and SAT (**I**) as well as Gal-12, ATGL and PPARγ in VAT (**M**) was evalated by qPCR, also at week 20. Data in panels H, I and M are presented as fold difference *versus* Lean WT mice after normalization for expression of GAPDH. Release of leptin (**K**) and APN (**L**) from cultures of VAT *ex vivo* was measured by ELISA. Data are expressed as pg of adipokine/mg of VAT. Data are mean+/−SEM, n = 10. *p<0.05, **p<0.01 *versus* respective WT group; °p<0.05, °°p<0.01, °°°p<0.001 *versus* respective Lean group; ∧p<0.05,∧p<0.001 vs WT Lean.

To evaluate whether Gal-3 deficiency affects body weight, Lean and DIO WT and Gal-3 KO mice were studied longitudinally. Two separate experiments were performed with comparable results, therefore data from the two studies are presented and analyzed together. Although Gal-3 KO mice were slightly but significantly smaller than WT mice at 8 weeks of age, the difference between chow-fed Lean WT and Lean Gal-3 KO mice was not significant at any of the later time points (Fig. 1A). When fed a HFD, both DIO WT and DIO Gal-3 KO mice became significantly heavier than their Lean counterparts starting at 4 weeks of feeding (12 weeks of age), with DIO Gal-3 KO mice having an accelerated growth during the last 2 weeks of feeding, resulting in their body weight being significantly higher than that of DIO WT mice at the 19 and 20 week time points (Fig. 1A). No significant differences in food intake were observed between WT and Gal-3 KO mice on either chow or HFD (Fig. 1B). Evaluation of body composition at the end of the experiment in 20-week-old mice demonstrated the presence of a significantly higher fat mass in both Lean and DIO Gal-3 KO mice compared to diet-matched WT groups both in terms of absolute fat mass (Fig. 1C) and as% of total body weight (% fat mass was 9.8+/−1.6, 25.6+/−2.2, 39.9+/−2.9 and 53.7+/−1.1 in Lean WT, Lean KO, DIO WT and DIO KO mice, respectively, n = 10). The increased fat mass of Gal-3 KO mice was confirmed by significantly higher levels of circulating leptin in 20-week-old Lean and DIO Gal-3 KO mice compared to their respective WT groups (Fig. 1D). There was also a trend towards bigger adipocyte size in both VAT and SAT of male Gal-3 KO compared to WT mice, although the difference did not reach statistical significance (Fig. 1 E–F). Significantly elevated circulating levels of triglycerides (TG) were present in DIO Gal-3 KO mice, with a non-significant trend in Lean Gal-3 KO mice compared to diet-matched WT groups (Fig. 1G). Moreover, significantly reduced mRNA expression of adiponectin (APN) was observed in VAT of both Lean and DIO Gal-3 KO mice compared to levels observed in Lean and DIO WT controls (Fig. 1H). On the other hand, as we previously demonstrated [13], a significant increase in mRNA expression for APN was observed in SAT of DIO WT mice compared to Lean WT mice, with APN expression in SAT of Lean and DIO Gal-3 KO mice comparable to that of DIO WT mice (Fig. 1I). Likely as a result of differential APN expression in VAT and SAT, circulating levels of APN were comparable in the four groups (Fig. 1J). Increased release of leptin and decreased production of APN from VAT of Gal-3 KO mice was confirmed *ex vivo* using adipose tissue cultures (Fig. 1K–L).

Evaluation of genes involved in adipose tissue metabolism indicated a reverse relationship with adiposity among the four groups, with significantly reduced expression of Gal-12 and adipose tissue triglyceride lipase (ATGL) in Gal-3 KO mice compared with diet-matched groups, as well as blunted expression of PPARγ in each group compared to Lean WT mice (Fig. 1M).

In summary, Gal-3 KO mice on either chow or HFD developed excess adiposity at 20 weeks of age compared to WT mice that was mirrored by measurement of biomarkers for adipose tissue metabolism.

### Liver phenotype of Lean and DIO Gal-3 KO mice

**Figure 2:**
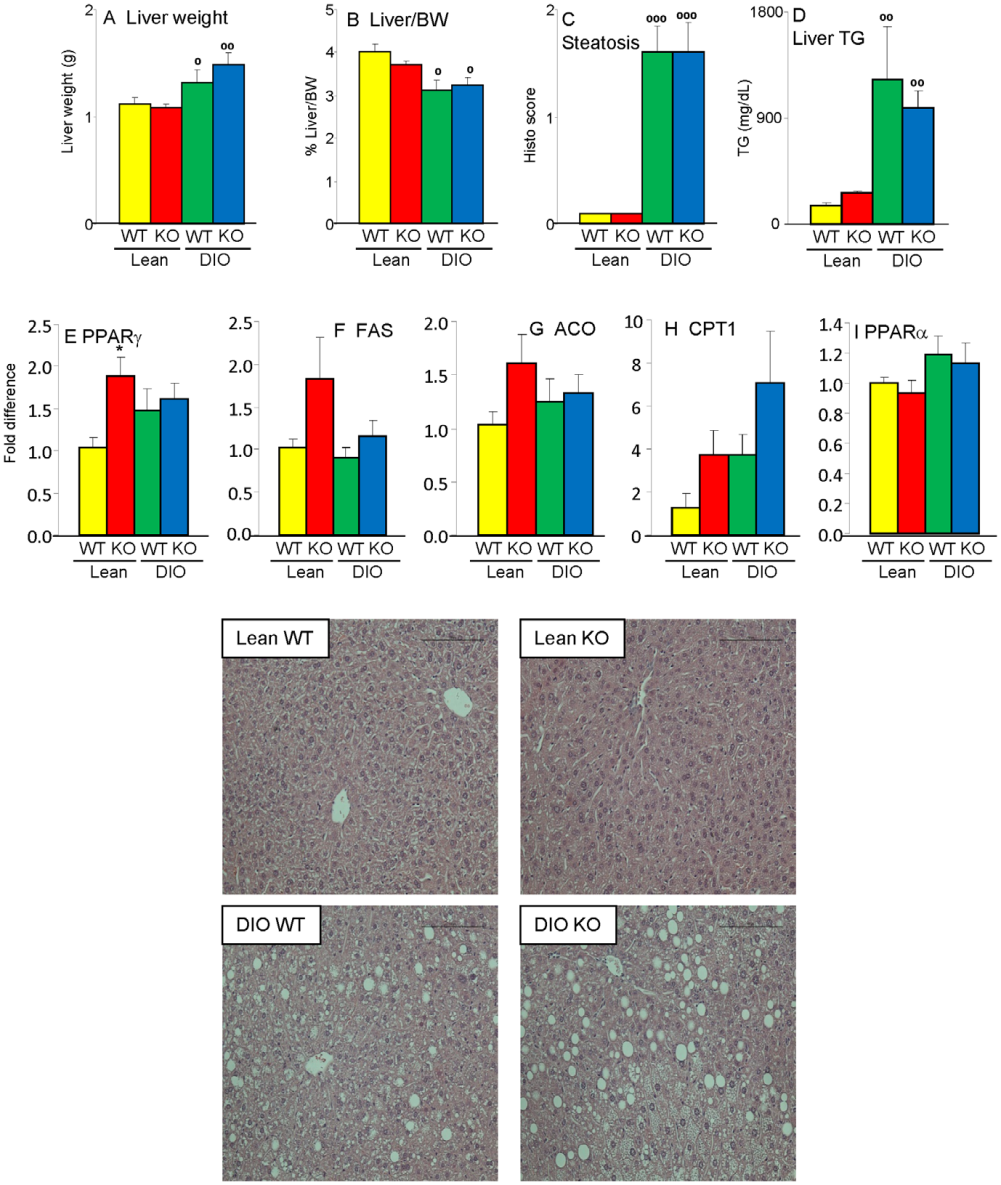
Hepatic steatosis in DIO WT and Gal-3 KO mice. Liver weight (**A**), ratio of liver/body weight (**B**), degree of hepatic steatosis (**C**), liver TG levels (**D**) as well mRNA expression of PPARγ (**E**), FAS (**F**), ACO (**G**), CPT1 (**H**) and PPARα (**I**) were evaluated in Lean WT (yellow), Lean Gal-3 KO (red), DIO WT (green) and DIO Gal-3 KO (blue) mice. Gene expression data are presented as fold difference *versus* Lean WT mice after normalization for expression of GAPDH. A representative H&E staining (20X) from each group is shown. Data are mean+/−SEM, n = 10. *p<0.05 *versus* respective WT group; °p<0.05, °°p<0.01, °°°p<0.001 *versus* respective Lean group.

Previous studies provided conflicting results on the effect of Gal-3 deficiency on development of hepatic steatosis [9], [11], [12]. We did not observe any significant difference in liver weight, ratio of liver/body weight or degree of hepatic steatosis between WT and Gal-3 KO mice, with each animal on HFD developing marked liver steatosis, with characteristics of mixed micro- and macrovesicular steatosis (Fig. 2A–C and representative pictures). Measurement of hepatic TG levels confirmed the results of histological analysis, with significantly elevated and comparable levels in both DIO WT and Gal-3 KO mice compared to lean groups (Fig. 2D). No histological signs of inflammatory infiltrate were observed in the liver regardless of diet or genotype (see pictures in Fig. 2). Furthermore, as shown below) hepatic mRNA expression of interleukin-6 (IL-6) was comparable in each group, confirming lack of overt liver-derived inflammation. Expression of genes involved in fat metabolism demonstrated a significantly increased expression of PPARγ in Lean Gal-3 KO *versus* Lean WT mice, with no significant differences between DIO groups (Fig. 2E), in agreement with previous reports [9], [12]. There was also a non-significant trend toward elevated expression of fatty acid synthase (FAS) and acyl-CoA oxidase (ACO) in Lean Gal-3 KO *versus* Lean WT mice (Fig. 2F–G). Moreover, we observed a non-significant trend for elevated expression of carnitin palmitoyltransferase 1 (CPT1) in both Gal-3 KO groups compared to diet-matched WT mice (Fig. 2H), with no effect of genotype on hepatic expression of PPARα (Fig. 2I). In summary, Gal-3 deficiency did not significantly affect the liver in terms of degree of steatosis or inflammation, although trends toward dysregulated expression of genes involved in fat metabolism were observed, particularly in Lean Gal-3 KO mice.

### Dysregulated glucose metabolism in Gal-3 KO mice

**Figure 3:**
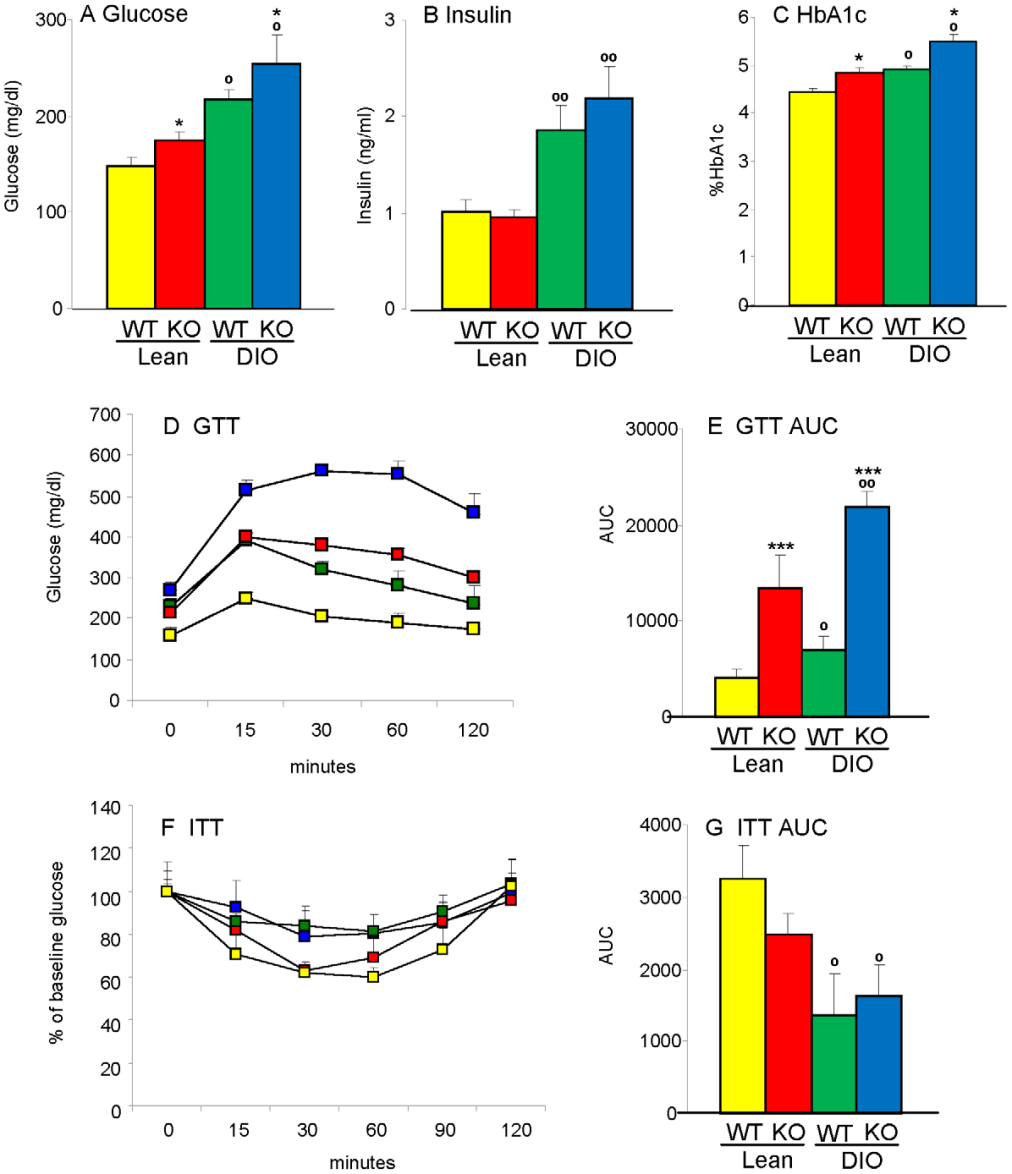
Glucose intolerance in Gal-3 KO mice. Parameters of glucose and insulin tolerance were evaluated in Lean WT (yellow), Lean Gal-3 KO (red), DIO WT (green) and DIO Gal-3 KO (blue) mice. Glucose (**A**), insulin (**B**) and% HbA1c (**C**) were evaluated in 4-hour fasted mice. Glucose tolerance test (**D**) was performed on 4-hour fasted mice. The area under the cruve for GTT is shown in **E**. Insulin tolerance test (**F**) was performed on fed mice. The area under the cruve for ITT is shown in G. Data are mean+/−SEM, n = 10 for A–C, n–5 for D–G. *p<0.05, ***p<0.001 *versus* respective WT group; °p<0.05, °°p<0.01 *versus* respective Lean group.

Significantly higher fasting glucose levels were observed in 20 week-old male Lean and DIO Gal-3 KO mice compared to age-matched Lean and DIO WT mice, with DIO groups of either genotype being hyperglycemic compared to Lean groups (Fig. 3A). However, fasting circulating levels of insulin were comparable between WT and KO mice (Fig. 3B). Calculation of the homeostatic model assessment index for insulin resistance (HOMA-IR) indicated the presence of insulin resistance in both DIO WT and Gal-3 KO mice compared to their respective Lean groups, with a non-significant elevation of the index in DIO Gal-3 KO *versus* DIO WT mice (the HOMA2 index was 3.1+/−0.3, 3.1+/−0.2, 5.7+/−0.5 and 8.5+/−1.3 in Lean WT, Lean Gal-3 KO, DIO WT and DIO Gal-3 KO mice, respectively. Data are mean+/−SEM of 9–10 mice per group; p<0.01 for DIO WT and DIO Gal-3 KO *versus* Lean WT and Lean KO mice). Moreover, both Lean and DIO Gal-3 KO mice had significantly higher levels of HbA1c compared to WT mice, with Lean Gal-3 KO mice having levels comparable to those of the DIO WT group (Fig. 3C).

A markedly exaggerated glycemic response to a glucose tolerance test (GTT) was observed in both Lean and DIO Gal-3 KO mice; the most severe glucose intolerance was observed in DIO Gal-3 KO mice (Fig. 3D–E). However, Gal-3 KO mice were as insulin-sensitive as their diet-matched WT controls as evaluated during an insulin tolerance test (ITT) (Fig. 3F–G).

**Figure 4:**
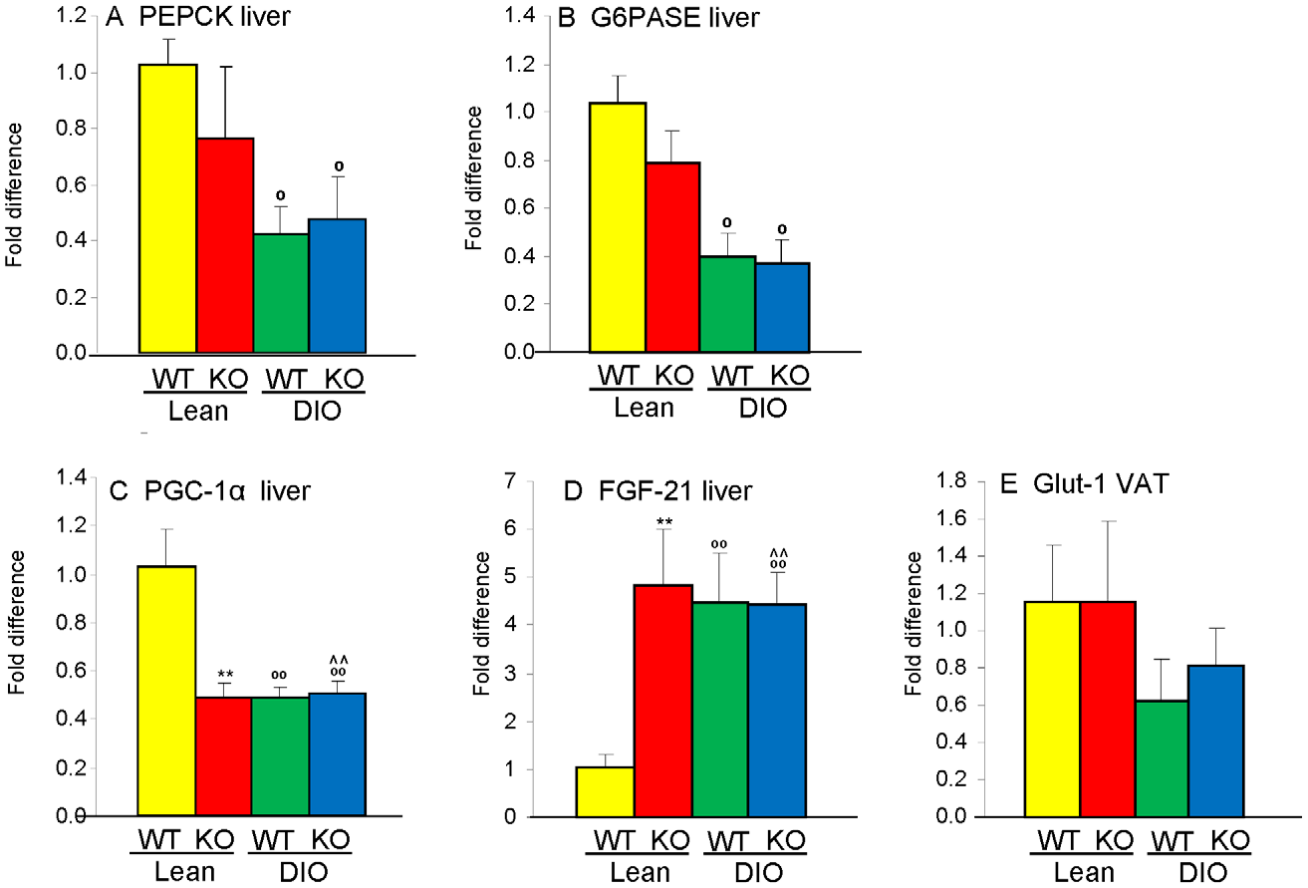
Expression of metabolic enzymes in WT and Gal-3 KO mice. Hepatic mRNA expression of PEPCK (**A**), G6PASE (**B**), PGC-1α (**C**) and FGF-21 (**D**), as well as expression of Glut-1 in VAT (**E**) was evaluated in Lean WT (yellow), Lean Gal-3 KO (red), DIO WT (green) and DIO Gal-3 KO (blue) mice. Data are expressed as fold difference *versus* Lean WT mice after normalization for expression of GAPDH. Data are mean+/−SEM, n = 10. **p<0.01 *versus* respective WT group; °p<0.05, °°p<0.01 *versus* respective Lean group; &∧p<0.05 vs WT Lean.

Impaired fasting glucose and inability to clear a glucose load may result from upregulated hepatic gluconeogenesis [16]. Therefore, to investigate the mechanisms leading to glucose intolerance in Gal-3 KO mice, we evaluated expression of genes involved in gluconeogenesis. Hepatic expression of the enzymes phosphoenolpyruvate carboxykinase (PEPCK) and glucose 6-phosphatase (G6PASE) was significantly reduced in fasted DIO mice compared to Lean mice, in agreement with previous results [17], with no significant differences due to genotype (Fig. 4A–B). Expression of PPARγ coactivator 1α (PGC-1α) - which controls genes involved in energy metabolism [18] -in the liver of Lean Gal-3 KO mice was approximately 50% lower than that of Lean WT mice and reached the same low level observed in DIO WT mice (Fig. 4C). Feeding a HFD to Gal-3 KO mice did not further reduce hepatic PGC-1α expression (Fig. 4C). A similar pattern, though in the opposite direction, was observed for hepatic expression of fibroblast growth factor-21 (FGF-21), a hormone that regulates carbohydrate and fatty acid metabolism [19]. In fact, expression of FGF-21 mRNA was significantly elevated in the liver of Lean Gal-3 KO mice, to the same extent observed in DIO WT and DIO Gal-3 KO mice (Fig. 4D). Stimulation of adipocytes with FGF-21 upregulates Glucose transporter-1 (Glut-1), leading to insulin-independent disposal of glucose to adipose tissue [20]. Thus, we speculated that reduced expression of Glut-1 might account for the defective clearance of glucose in the presence of maintained insulin sensitivity of Gal-3 KO mice. However, expression of Glut-1 in VAT did not significantly differ between WT and Gal-3 KO mice (Fig. 4E).

To summarize, Gal-3 deficiency was associated with inability to promptly clear a glucose load in the presence of preserved insulin sensitivity, without significant alterations in expression of major gluconeogenic enzymes. Furthermore, expression of genes involved in fatty acid oxidation and glucose disposal in Lean Gal-3 KO mice mirrored the pattern observed in DIO mice.

### Inflammation in Gal-3 KO mice

**Figure 5:**
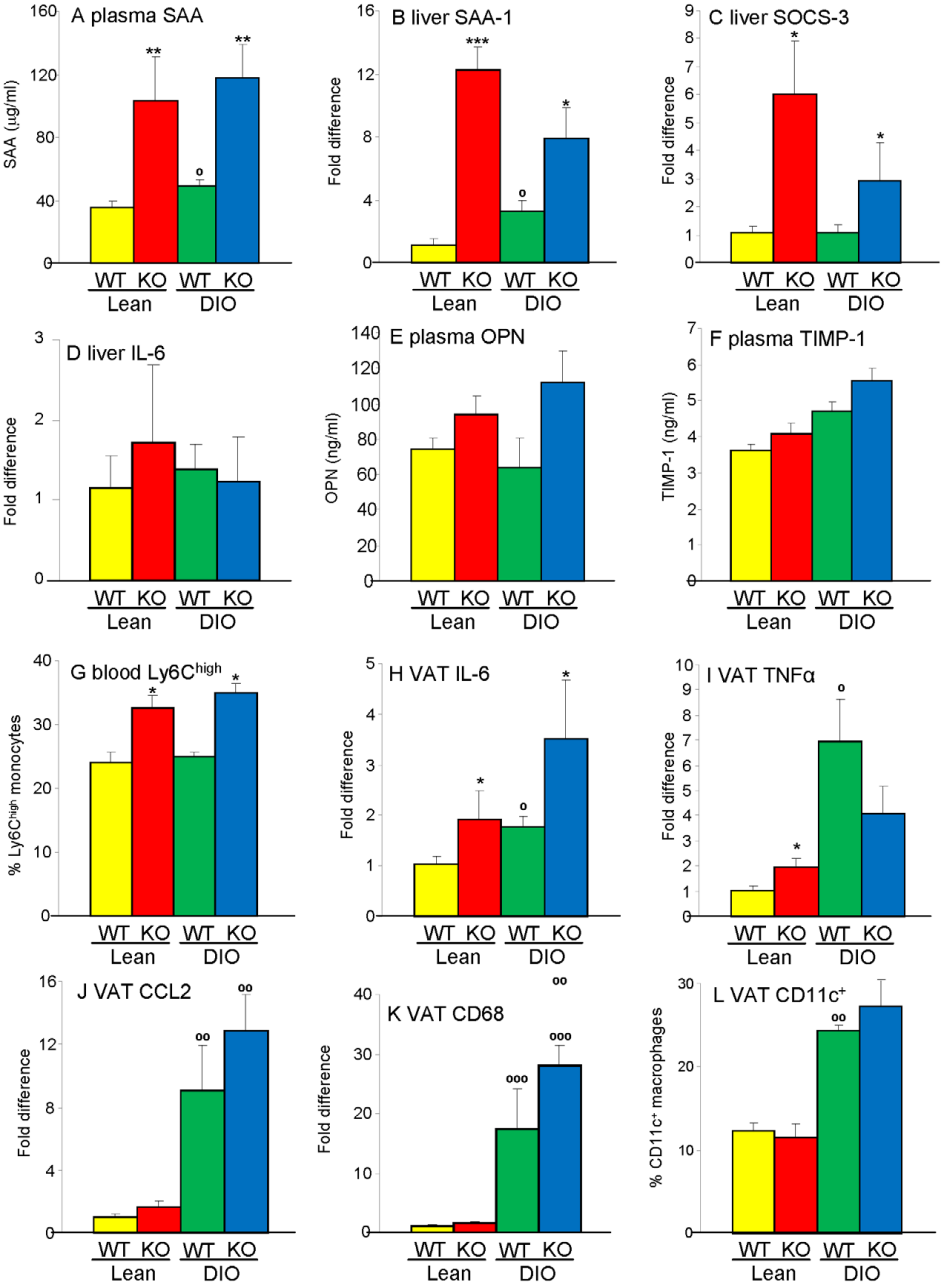
Systemic and adipose tissue inflammation in Gal-3 KO mice. Inflammation was evaluated by measuring plasma levels of SAA (**A**), hepatic mRNA expression of SAA-1 (**B**), SOCS-3 (**C**), and IL-6 (**D**), plasma levels of OPN (**E**) and TIMP-1 (**F**) as well as% of circulating Ly6C^high^ cells (**G**) in Lean WT (yellow), Lean Gal-3 KO (red), DIO WT (green) and DIO Gal-3 KO (blue) mice. Gene expression of IL-6 (**H**), TNFα (**I**), CCL2 (**J**), CD68 (**K**) as well as% of infiltrating F4/80^+^/CD11c^+^ macrophages (**L**) in VAT were used as markers of adipose tissue inflammation. Data for mRNA expression are presented as fold difference *versus* Lean WT mice after normalization for expression of GAPDH. Data are mean+/−SEM, n = 10. *p<0.05, **p<0.01, ***p<0.001 *versus* respective WT group; °p<0.05, °°p<0.01, °°°p<0.001 versus respective Lean group.

A significant elevation in circulating levels and hepatic mRNA expression of the acute-phase protein serum amyloid A (SAA) was observed in Lean and DIO Gal-3 KO mice compared to their WT counterparts (Fig. 5A–B). This was paralleled by elevated expression of suppressor of cytokine signaling-3 (SOCS-3), a proxy for activated STAT-3 [21], in the liver of Gal-3 KO compared with WT mice, particularly in Lean groups (Fig. 5C). However, as mentioned above, hepatic expression of IL-6 was not significantly different among groups (Fig. 5D), suggesting an extra-hepatic source of inflammation. Trends towards elevated circulating levels of osteopontin (OPN) and tissue inhibitor of metalloprotease-1 (TIMP-1) [22] in Gal-3 KO mice confirmed the presence of systemic inflammation (Fig. 5 E–F).

The association of Gal-3 deficiency with systemic inflammation was also supported by development of neutrophilia, microcytic anemia and thrombocytemia in both Lean and DIO Gal-3 KO mice compared with their WT controls (Table 1). Despite lack of differences in the absolute number and percentage of monocytes in peripheral blood, the proportion of Ly6C^high^ monocytes (Ly6G-Ly6B^+^Ly6C^high^ cells) in blood, a cell type with pro-inflammatory activity [23], was significantly higher in Gal-3 KO compared to WT mice irrespective of diet (Fig. 5G).

**Table 1:** Hematological parameters in Lean and DIO WT and Gal-3 KO mice.

	Lean WT	Lean Gal-3 KO	DIO WT	DIO Gal-3 KO
WBC (10^3^/µl)	9.47+/−0.48	9.79+/−1.11	10.72+/−0.68	12.18+/−0.98
# NE (10^3^/µl)	1.68+/−0.15	2.53+/−0.49	2.21+/−0.21	2.86/−0.29
# LY (10^3^/µl)	7.43+/−0.40	6.80+/−0.58	7.94+/−0.63	8.63+/−0.56
# MO(10^3^/µl)	0.28+/−0.03	0.40+/−0.12	0.28+/−0.04	0.31+/−0.06
# EO (10^3^/µl)	0.06+/−0.03	0.05+/−0.03	0.05+/−0.01	0.06+/−0.02
# BA (10^3^/µl)	0.02+/−0.01	0.02+/−0.01	0.01+/−0.00	0.01+/−0.00
%NE	17.68+/−1.34	24.65+/−2.69*	21.21+/−1.08	25.28+/−1.32*
%LY	78.49+/−1.61	71.21+/−3.19	75.50+/−1.46	71.77+/−1.51
%MO	2.94+/−0.27	3.54+/−0.82	2.69+/−0.28	2.44+/−0.34
%EO	0.69+/−0.32	0.47+/−0.29	0.49+/−0.11	0.45+/−0.15
%BA	0.19+/−0.10	0.15+/−0.10	0.11+/−0.05	0.07+/−0.02
RBC (10^6^/µl)	9.25+/−0.10	9.59+/−0.19	10.01+/−0.25#	10.42+/−0.21#
Hb (g/dl)	13.52+/−0.16	11.85+/−0.35*	14.32+/−0.31	12.79+/−0.23*
Hct	44.83+/−0.45	36.24+/−0.93*	47.86+/−1.35	41.20+/−0.59*
MCV (fl)	48.50+/−0.47	37.83+/−0.67*	47.76+/−0.40	35.59+/−0.65*
MCH (pg/cell)	14.64+/−0.12	12.36+/−0.25*	14.31+/−0.15	12.28+/−0.17*
MCHC (g/dl)	31.06+/−0.26	32.54+/−0.17	31.00+/−0.13	31.42+/−0.15
RDW (%)	17.56+/−0.14	19.72+/−0.34*	18.62+/−0.10	21.14+/−0.29*
Plat (10^3^/µl)	895+/−65	1309+/−51*	756+/−27	1009+/−50*
MPV (fl)	4.69+/−0.14	4.61+/−0.07	4.55+/−0.08	4.75+/−0.07

White blood cell (WBC) counts, Neutrophil (NE), Lymphocyte (LY), Monocyte (MO), Eosinophil (EO) and Basophil (BA) absolute numbers and percentages, Red Blood cell (RBC) numbers, Hemoglobin (Hb) levels, Hematocrit (Hct), Mean corpuscular volume (MCV), Mean corpuscular hemoglobin (MCH), Mean corpuscular hemoglobin concentration (MCHC), Red cell Distribution Width (RDW), platelet (Plat) number and Mean Platelet Volume (MPV) were evaluated on EDTA-anticoagulated peripheral blood using a Hemavet 950FS. Data are mean+/−SEM of 8–10 mice per group. *p<0.05 vs respective WT group; °p<0.05 vs respective Lean group.

Evaluation of expression of inflammatory markers in VAT demonstrated significantly elevated expression of IL-6 in both Lean and DIO Gal-3 KO mice compared to the respective WT mice (Fig. 5H). Lean Gal-3 KO mice also had significantly higher expression of tumor necrosis factor α (TNFα) *versus* Lean WT mice, although this difference was not observed in DIO groups (Fig. 5I). On the other hand, only a non-significant trend towards elevated expression of the chemokine C–C motif ligand 2 (CCL2) and the marker of macrophage infiltration CD68 was observed in Lean and DIO Gal-3 KO mice compared to WT mice (Fig. 5J–K). Flow cytometry analysis of VAT-infiltrating cells demonstrated the presence of comparable percentages of pro-inflammatory F4/80^+^CD11c^+^ macrophages in VAT of WT and Gal-3 KO mice, with DIO groups having significantly higher F4/80^+^CD11c^+^ cells compared to Lean groups, as expected [24] (Fig. 5L). In summary, systemic inflammation was present in 20-week-old male Gal-3 KO mice irrespective of diet. The source of the inflammatory response was, at least in part, located in VAT and targeted the liver and hematopoietic system.

### Dysregulated glucose metabolism without excess adiposity or inflammation in young Gal-3 KO mice

**Figure 6:**
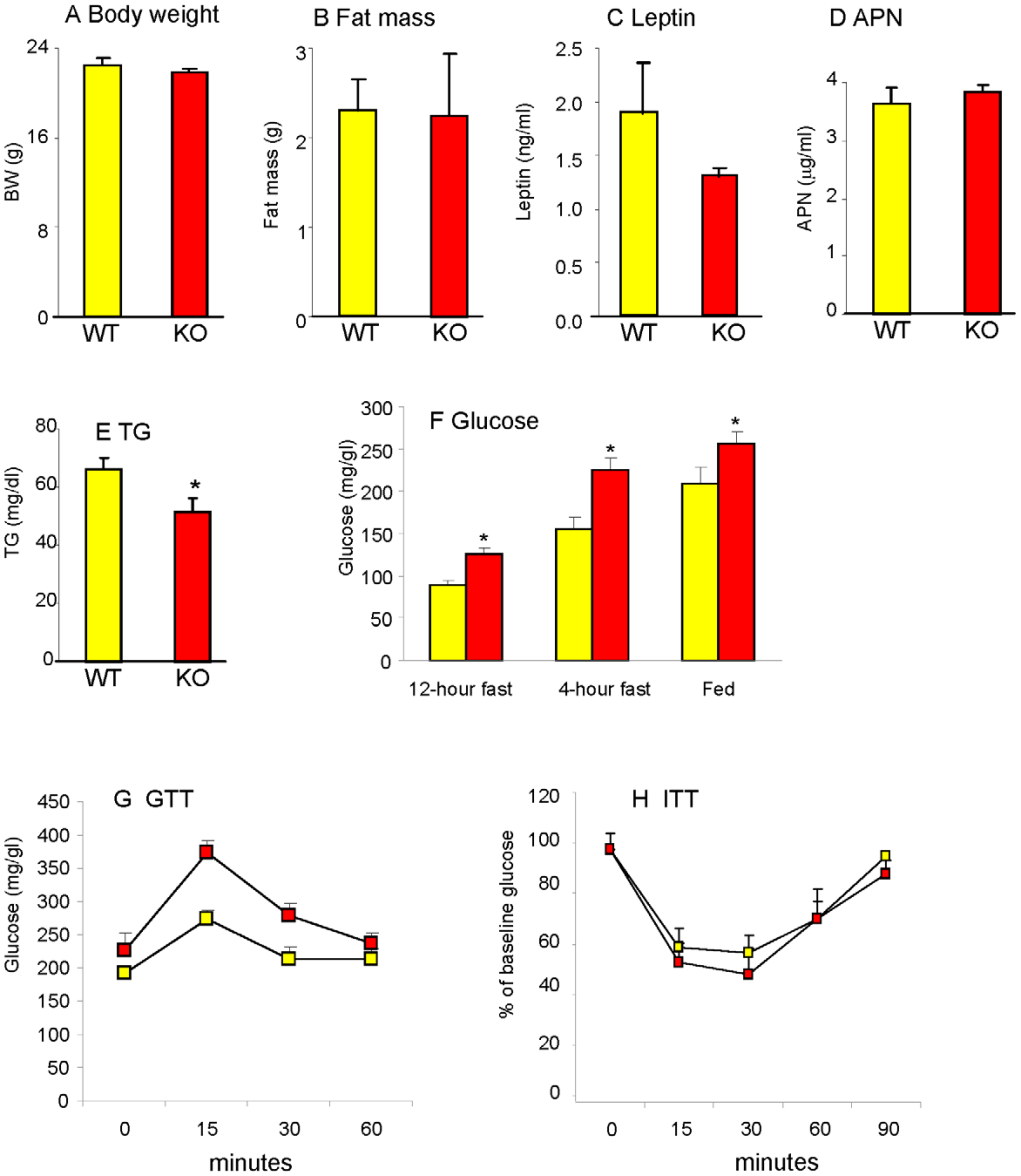
Adiposity and glucose metabolism in young Gal-3 KO mice. Body weight (**A**), fat mass (**B**), plasma levels of leptin (**C**), APN (**D**) and TG (**E**) as well as blood levels of glucose (**F**) were evaluated in chow-fed 12-week-old WT (yellow) and Gal-3 KO (red), mice. Glucose tolerance test (**G**) was performed on 4-hour fasted mice. Insulin tolerance test (**H**) was performed on fed mice. Data are mean+/−SEM, n = 5. *p<0.05 *versus* WT mice.

Due to the close interrelationship between adiposity, glucose metabolism and inflammation [25] and in order to begin clarifying cause-effect relationship leading to the phenotype we observed in 20-week-old Gal-3 KO mice, a separate set of experiments was performed in chow-fed 12-week old male mice. As shown in Figure 6A–D, no significant differences in body weight, fat mass, serum leptin or APN were observed between 12-week-old male WT and Gal-3 KO mice, with these latter having significantly lower circulating TG levels compared to WT mice (Fig. 6E). However, significantly higher blood glucose levels were already evident at this age in male mice, with mild hyperglycemia being present irrespective of fasting or fed state (Fig. 6F). Significantly higher fasting glucose levels were also observed in 12-week-old female Gal-3 KO *versus* WT mice (71.0+/−5.6 *versus* 102.3+/−4.7 mg/dl in WT and Gal-3 KO female mice, respectively; p = 0.013, n = 3). Impaired glucose tolerance with maintained insulin sensitivity was also already evident in 12-week-old male Gal-3 KO mice, as indicated by GTT (Area under the curve was 4458+/−386 vs 7507+/−675 in WT and Gal-3 KO mice, respectively; p = 0.004, n = 5) and ITT (Area under the curve was 2358+/−436 vs 2746+/−669 in WT and Gal-3 KO mice, respectively, n = 5) (Fig. 6G–H). No significant differences in expression of PEPCK, PGC-1α, FGF-21 or SAA-1 in liver as well as APN, CCL2, CD68 or IL-6 in VAT were observed between 12-week-old male Gal-3 KO and WT mice (not shown). Furthermore, 12-week-old male Gal-3 KO mice did not display neutrophilia compared to age-matched WT mice (% of neutrophils in blood was 16.3+/−0.5 and 15.3+/−1.1 in WT and Gal-3 KO mice, respectively; n = 5). Thus, alterations in glucose metabolism precede development of excess adiposity and inflammation in Gal-3 KO mice.

### Effect of antibiotic treatment on glucose control in young Gal-3 KO mice

**Figure 7:**
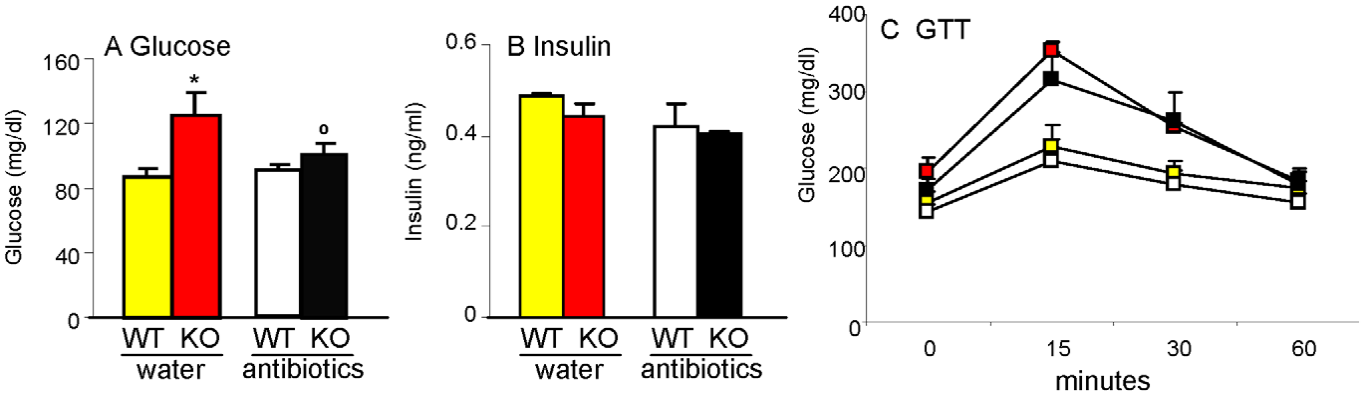
Effect of treatment with antibiotics. Chow-fed 8-week-old WT and Gal-3 KO mice received either regular drinking water (WT: yellow; Gal-3 KO: red) or water supplemented with antibiotics (WT: white; Gal-3 KO: black) for 4 weeks before measurement of fasting glucose (**A**), fasting insulin (**B**) and response to GTT (**C**) Data are mean+/−SEM, n = 5. *p<0.05 *versus* respective WT; °p<0.05 *versus* respective group without antibiotics (water).

To investigate the potential role of the microbiota in mediating the effect of Gal-3 deficiency on glucose metabolism, 8-week-old WT and Gal-3 KO mice received either regular water or water supplemented with antibiotics for 4 weeks, following a previously described protocol that successfully normalized glucose metabolism by sterilizing the gastrointestinal tract in TLR2 KO mice [26]. Antibiotic treatment significantly reduced the elevated fasting glucose levels of Gal-3 KO mice without affecting glucose levels in WT mice (Fig. 7A) or insulin levels in either group (Fig 7B). However, gut sterilization did not ameliorate the excessive response to GTT of Gal-3 KO mice (Fig. 7C). Thus, sterilization of the gut normalized fasting glucose levels in young Gal-3 KO mice, but did not alter the inability of Gal-3 KO mice to properly dispose a glucose load.

## Discussion

In the present report we demonstrate that Gal-3 deficiency in mice leads to dysregulated glucose metabolism as well as age-related excess adiposity and inflammation. Studies performed in 12-week-old Gal-3 KO mice indicate that altered glucose metabolism precedes accumulation of adipose tissue and development of inflammation. Moreover, the data point to a potential role of the microbiota in mediating hyperglycemia in Gal-3 KO mice.

Young Gal-3 KO mice developed mild hyperglycemia and were unable to effectively clear glucose from the circulation during a glucose load, in the absence of obesity or systemic inflammation. This response was exacerbated in older Gal-3 KO mice, especially when fed a HFD. The presence of chronically elevated glucose levels in Gal-3 KO mice was confirmed by measurement of HbA1c, a measure of glycemic control over the life course of erythrocytes. The impaired glucose tolerance of Gal-3 KO mice was not due to development of more severe insulin resistance compared to WT mice, as assessed by ITT. Therefore, we hypothesized that Gal-3 deficiency might be associated with an excessive gluconeogenic response and/or with inability to properly activate insulin-independent disposal of glucose to adipose tissue through upregulation of Glut-1. However, we found that expression of gluconeogenic enzymes in the liver and of Glut-1 in VAT was not altered in Gal-3 KO mice compared to WT groups, suggesting other mechanisms of action, possibly including modulation of glucagon production and/or glucose uptake by muscle [27].

Normalization of blood glucose levels by antibiotics pointed to a role for the microbiota in mediating the mild hyperglycemia of Gal-3 KO mice. This is in agreement with studies demonstrating an important role for the gut microflora in modulating glucose metabolism, at least in part through activation of pattern recognition receptors [2], [26]. However, gut sterilization did not ameliorate the exaggerated response to a glucose load of Gal-3 KO mice. This suggests that other mechanisms modulate this response, including perhaps the ability of Gal-3 to act as a receptor and increase clearance of glucose adducts [5]. Our data on glucose control are at variance with studies reporting normal glucose and HbA1c levels in Gal-3 KO mice [6], [8], [9]. Given the potential role of the microbiota in mediating the effect of Gal-3 deficiency on glucose metabolism, the influence of housing conditions and microbial environment may explain the discrepancy between our findings and these previous results. Another potential explanation for discrepancies with previous studies is the age-dependent difference in circulating TG levels between WT and Gal-3 KO mice.

By 20-weeks of age Lean and DIO Gal-3 KO mice had accumulated significantly more adipose mass and had higher systemic leptin levels compared with their respective WT mice. Since the increased adiposity of Gal-3 KO mice cannot be attributed to increased food intake, either reduced energy expenditure or more efficient utilization of calories should explain the findings. Both Gal-3 deficiency and DIO were associated with downregulation of PGC-1α expression in the liver, in agreement with previous studies indicating that DIO leads to reduced hepatic PGC-1α levels [18]. Reduced expression of PGC-1α was associated with elevated FGF-21, as expected based on the suppressive effect of PGC-1α on hepatic FGF-21 [28]. Furthermore, expression of APN, Gal-12, ATGL, PPARγ, IL-6 and TNFα in VAT of Lean Gal-3 KO mice mirrored the levels observed in DIO WT mice. In particular, reduced expression of ATGL may participate in the increased adiposity of Gal-3 KO mice, since this enzyme plays a critical role in catabolism of stored fat [29]. However, the study design did not allow us to discern whether altered expression of any of these genes is a cause or an effect of the increased adiposity of Gal-3 KO mice. Future studies aimed at characterizing cause-effect relationships are necessary to clearly delineate the pathway leading to development of age-related obesity in Gal-3 KO mice. This is particularly important in light of the elevation of Gal-3 production observed in obese subjects [10] and of the current advanced development of pharmacological inhibitors of Gal-3 for treatment of cancer and fibrosis.

Despite the presence of significantly increased adiposity in Gal-3 KO mice, adipocytes were not significantly larger than those of diet-matched WT mice. This finding can potentially be attributed to lack of Gal-3 and significantly reduced production of Gal-12 as well as PPARγ, in Gal-3 KO mice, since all of these factors have been implicated in adipogenesis and adipocyte differentiation [15], [30].

Our results also demonstrate development of age-related systemic inflammation in Gal-3 KO mice, irrespective of diet. This was associated with elevated production of the acute-phase protein SAA, hepatic expression of SOCS-3 as well as development of neutrophilia and other hematological alterations. Induction of SAA in obesity is mediated by IL-6 [31]. Because we could not detect elevated expression of IL-6 in the liver of Gal-3 KO mice compard to diet-matched WT groups, data suggest an extra-hepatic source for this cytokine. Indeed, significantly higher expression of IL-6 was present in VAT of Gal-3 KO mice compared with WT controls, indicating that adipose tissue might be at least partly responsible for induction of the STAT-3 target genes SOCS-3 and SAA in the liver [21] However, caution should be employed in intepreting data on elevated hepatic expression of SOCS-3 in the context of the metabolic phenotype of Gal-3 KO mice, since liver-specific deletion of this transcription factor leads to fatty liver and obesity in the presence of enhanced hepatic insulin sensivity [32]. Our results on the inflammatory state of Gal-3 KO mice are in agreement with studies demonstrating exacerbated inflammation in Gal-3 KO mice in response to endotoxin or metabolic stress [4]-[8], [11], [12], [33], as well as with data pointing to increased expression of inflammatory cytokines in macrophages from Gal-3 KO mice [34]. The ability of Gal-3 to promote polarization of macrophages towards the anti-inflammatory M2 phenotype, to clear endotoxin, promote phagocytosis of apoptotic cells and participate in clearance of pro-inflammatory glucose and lipid adducts may contribute to explain development of age-related inflammation in the absence of Gal-3 [4], [35]–[37] However, our data apparently contradict clinical results demonstrating a positive correlation between circulating levels of Gal-3 and markers of inflammation [38]. Evidence provided in the current report together with previous experimental results suggest that increased production of Gal-3 during inflammation may represent an attempt at dampening excessive chronic inflammatory responses, even though the complex regulation of intra- and extra-cellular compartmentalization of Gal-3 as well as potential modulation of Gal-3 receptors and/or ligands in the presence of HFD requires more detailed studies.

Our study has several limitations, including lack of evaluation of the possible role of muscle in modulating glucose uptake, lack of measurement of metabolic enzymes at the protein level, as well as use of non-littermate control groups.

In conclusion, we demonstrate that Gal-3 plays an important role in modulating glucose metabolism in mice, in part through the microbiota. The mild hyperglycemia and inability to handle a glucose load of young Gal-3 KO mice is followed by development of excess adiposity and systemic inflammation as animals age and is compounded by high-fat feeding. Since elevated Gal-3 is present in humans with obesity, diabetes, heart failure and other diseases associated with inflammation [10], [38]–[40], these results may contribute to a better understanding of the role Gal-3 plays in these conditions.

## Materials and Methods

### Ethics statement

Animal studies were approved by the Animal Care and Use Committee of the University of Illinois at Chicago under protocol A10-008.

### Animals

Male WT and Gal-3 KO mice (B6.Cg-*Lgals3^tm1Poi^*/J) on a C57BL6 background were obtained from The Jackson Laboratories. Animals of the two strains were age-matched but were not littermates. For induction of DIO, mice were fed a HFD (60 Kcal% fat, 7% Kcal/fructose, Research Diets) *ad libitum* for 12 weeks beginning at 8 weeks of age, while Lean groups received standard chow diet. Body composition was evaluated by dual energy X-ray absorbtiometry (DXA) at time of euthanasia. Blood was collected in EDTA tubes. After evaluation of hematologic parameters using the HV950FS (Drew Scientific), 50 μl of blood were used for flow cytometry analysis as detailed below, while the remaining blood was centrifuged and plasma obtained and stored at −70°C for subsequent analysis. The liver was weighed and portions fixed in formalin for histological analysis and frozen in liquid nitrogen for gene expression studies. Portions of epidydimal VAT and of SAT were fixed in formalin for histological analysis and frozen in liquid nitrogen for gene expression. A second experiment with the same study design was performed to confirm results. In a separate experiment, male and female WT and Gal-3 KO mice fed chow diet were studied at 12 weeks of age. Finally, 8-week-old male WT and Gal-3 KO mice fed chow diet received either regular drinking water or water supplemented with broad spectrum antibiotics (1 g/L ampicillin, 1 g/L metronidazole, 0.5 g/L neomycin) for 4 weeks, following a previously described protocol aimed at sterilizing the gastrointestinal tract [26].

### Glucose and insulin tolerance tests and calculation of insulin resistance

For GTT, mice were fasted for 4 hours before receiving an ip injection of 1 g/kg of dextrose. For ITT, fed mice received an ip injection of 1 IU/Kg of insulin. Blood glucose levels were measured from a tail nick immediately before injection of glucose or insulin and at various times thereafter. The Homeostatic Model of Insulin Resistance (HOMA) 2 index was determined using the calculator available ar www.dtu.ox.ac.uk/homacalculator/, which is based on [41].

### Measurement of adipocyte size and liver steatosis

Formalin-fixed portions of VAT, SAT and liver were sectioned at 5 µm and stained with hematoxilin-eosin for analysis. For measurement of adipocyte size in VAT and SAT the software Image J was used. Specifically, surface area in µm^2^ was measured from a total of 100 cells per mouse in three separate fields and the median value calculated for each tissue in each animal. An operator blinded to the experimental group performed the analysis. Scores for livers steatosis were calculated by a pathologist blinded to the treatment group using the following scale: 0 = <5% hepatocytes affected; 1 = 5–33% hepatocytes affected; 2 = 34–66% hepatocytes affected; 3 = >67% hepatocytes affected. Evaluation of the type of steatosis (predominantly microvesicular, predominantly macrovesicular or mixed microvesicular and macrovesicular) was also performed. Finally, histological presence of inflammation was scored as follows: 0 = <1 focus per 20X field; 1 = 1–2 foci per 20X field; 2 = 3–4 foci per 20X field; 3 = >4 foci per 20X field.

### Adipose tissue culture

A piece of ∼50 mg of VAT was cultured for 24 hours in 24-well plates in RPMI containing penicillin and streptomycin. The supernatant was collected and frozen before measurement of leptin and APN by ELISA.

### Separation of adipocytes from the stromo-vascular fraction

Collagenase digestion was used to separate the stromo-vascular fraction from adipocytes of epididymal VAT obtained from male 20-week-old Lean and DIO mice WT and Gal-3 KO mice, as previously described [42]. Cells from the stromo-vascular fraction were used for flow cytometry analysis as described below.

### Flow cytometry

Cells from the stromo-vascular fraction were resuspended in PBS/BSA prior to surface staining with anti-F4/80 (eBioscience) and anti-CD11c (BD Biosciences) antibodies. Whole blood was surface-stained with antibodies directed against Ly6C, Ly6G and Ly6B (BD Biosciences). Following lysis of erythrocytes, samples were analyzed on a C6 Accuri cytometer (BD Biosciences).

### Measurement of biomarkers

Blood glucose was measured using a glucometer and plasma insulin by ELISA (Alpco). Levels of HbA1c were measured using A1cNow+ (Bayer). Levels of leptin, APN, IL-6, SAA, OPN and TIMP-1 were measured using ELISA kits from R&D Systems or Life Technologies. Levels of TG were measured using a colorimetric assay from Cayman Chemicals.

### RNA expression analysis

Total RNA was isolated from liver, epididymal VAT and SAT using Trizol and reverse transcribed. Gene expression of ACO, APN, ATGL, CCL2, CD68, CPT1, FAS, FGF-21, Gal-12, G6PASE, Glut-1, IL-6, PEPCK, PGC-1α, PPARα, PPARγ, SAA-1, SOCS-3, and TNFα was assessed by real-time RT-PCR using the TaqMan system and primers from Applied Biosystems (Foster City, CA). Relative expression was calculated using the ΔΔ^CT^ method after normalizing for expression of GAPDH.

### Statistical analysis

Data are expressed as mean+/−SEM. Statistical significance of differences was evaluated by ANOVA. Log transformed values were used when data were not normally distributed. Statistical analyses were performed with MedCalc (Mariakerke, Belgium).
